# Integrating Deep Learning and Transcriptomics to Assess Livestock Aggression: A Scoping Review

**DOI:** 10.3390/biology14070771

**Published:** 2025-06-26

**Authors:** Roland Juhos, Szilvia Kusza, Vilmos Bilicki, Zoltán Bagi

**Affiliations:** 1 Centre for Agricultural Genomics and Biotechnology, University of Debrecen, 4032 Debrecen, Hungary; juhosroland@mailbox.unideb.hu (R.J.); kuszasz@hotmail.com (S.K.); 2 Doctoral School of Animal Science, University of Debrecen, 4032 Debrecen, Hungary; 3 Department of Software Engineering, University of Szeged, 6720 Szeged, Hungary; bilickiv@inf.u-szeged.hu

**Keywords:** livestock, behavior, aggressive, transcriptomics, computer vision, neuroethology

## Abstract

The presence of aggressive behavior in livestock creates major difficulties for animal welfare, farm safety, economic performance and selective breeding. The two innovative tools of deep learning-based video analysis and transcriptomic profiling have recently appeared to aid the understanding and monitoring of such behaviors. This scoping review assesses the current use of these two methods for aggression research across livestock species and identifies trends while revealing unaddressed gaps in existing literature. A scoping literature search was performed through the PubMed, Scopus and Web of Science databases to identify articles from 2014 to April 2025. The research included 268 original studies which were divided into 250 AI-driven behavioral phenotyping papers and 18 transcriptomic investigations without any studies combining both approaches. Most research focused on economically significant species, including pigs and cattle, yet poultry and small ruminants, along with camels and fish and other species, received limited attention. The main developments include convolutional neural network (CNN)-based object detection and pose estimation systems, together with the transcriptomic identification of molecular pathways that link to aggression and stress. The main barriers to progress in the field include inconsistent behavioral annotation and insufficient real-farm validation together with limited cross-modal integration. Standardized behavior definitions, together with multimodal datasets and integrated pipelines that link phenotypic and molecular data, should be developed according to our proposal. These innovations will speed up the advancement of livestock welfare alongside precision breeding and sustainable animal production.

## Simple Summary

Farm animal welfare and productivity, along with safety, depend on the proper understanding and management of aggressive behavior. Scientists have developed two contemporary methods to study aggression through smart video monitoring systems that analyze animal behaviors and which conduct brain gene expression analysis during aggressive states. The review examined 268 studies from 2014 to 2025 which included 250 artificial intelligence (AI)-based behavior monitoring studies and 18 transcriptomics studies but no studies that combined both methods. The review examined how these methods have been implemented across different farm species, including pigs, cows and chickens, but discovered that these tools remain disconnected. Most research continues to focus on well-known species while other species have received limited scientific attention. The review focuses on the requirement for standardized behavioral definitions together with affordable monitoring tools and multi-species research, particularly in practical farm settings. Scientists and farmers can better predict and prevent aggression through the combination of behavioral video data with genetic insights. An integrated approach would promote better animal health while ensuring farm safety and enhanced breeding results and sustainable livestock operations.

## 1. Introduction

Behavior and aggression in livestock are key elements of welfare, productivity and sustainability in animal husbandry [1]. Inimical interactions can lead to injuries, chronic stress, reduced feed intakes and reproductive failures, emphasizing the need for effective monitoring and early detection structures. These can create ethical problems which also endanger the long-term viability and efficiency of livestock production systems. The growing worldwide need for animal protein creates additional stress on farms to detect and prevent negative social behaviors in order to maintain both welfare and performance [2]. In recent years, the merging of precision livestock farming with AI-based phenotyping tools and transcriptomics has enabled remarkable insights into both external behavioral cues and internal regulatory mechanisms, though the combination of the two areas has yet to be explored [3,4].

Behavior and social stress evaluation in the past has depended on manual observations through ethograms and simple sensor-based monitoring systems, which have proved time-consuming and subjective, with limited resolution and scalability. Precision livestock farming (PLF) has brought automated monitoring tools for continuous non-invasive animal observation. Deep learning algorithms in computer vision systems have demonstrated exceptional effectiveness for detecting specific behavioral traits, including aggression, mounting, feeding and locomotion. Many systems employ CNNs and recurrent neural networks (RNNs) and pose estimation frameworks to detect minor postural and interaction patterns in video streams, which enables real-time large-scale phenotyping [5,6].

Meanwhile, transcriptomic technologies have revealed molecular pathways controlling social behavior and aggression, providing understanding of these complex attributes. The advancement of transcriptomic technologies including RNA sequencing (RNA-Seq) has enabled a better understanding of behavioral molecular mechanisms, including aggression, stress response and social hierarchy. Research on model and farm species has revealed gene networks that control neuroendocrine regulation, neurotransmission, inflammation and hormonal responses, which help scientists connect behavioral phenotypes to genomic pathways [7]. The application of transcriptomics enables researchers to discover biomarkers and develop predictive breeding methods with personalized welfare interventions for farm settings [8].

The two powerful methods of AI-based behavioral analysis and transcriptomics exist independently in the scientific literature. The fast development of both fields has not been accompanied by sufficient research that merges real-time behavioral information with molecular data, thus creating a significant void in computational neuroethology. The majority of current research has focused on economically dominant species including pigs and cattle, though small ruminants and poultry as well as aquaculture species and camels remain underrepresented despite their worldwide significance [9]. The current scoping review assesses the application of AI-assisted computer vision and transcriptomic techniques for livestock aggression evaluation while identifying species-specific research patterns and methodological challenges in uniting behavioral phenotyping with omics-level data. This paper examines recent developments and limitations and future paths for building integrated, scalable, and ethically grounded behavioral monitoring systems in precision livestock farming.

## 2. Materials and Methods

### 2.1. Databases Search Strategy

According to the PRISMA-ScR guideline, a literature search was performed on the following platforms: PubMed, Scopus and Web of Science. Searches were conducted in April and May 2025 for papers published between 2014 and 2025.

The used keywords were selected methodically after initial test searches, to identify the adequate prompts and Boolean operators. The accepted keywords with the Boolean operators were as follows: aggression AND RNA-Seq; aggression AND transcriptomics; behavior AND deep learning; behavior AND livestock AND transcriptomics; computational neuroethology; livestock AND aggression AND deep learning; livestock AND CNN; livestock AND pose estimation; livestock AND video analysis; transcriptomics AND deep learning AND behavior. This resulted in 10 advanced searches on each platform.

### 2.2. Selection Criterias, Selection Process

The inclusion of articles in the final literature pool were based on original research articles, in English only. During advanced and refined searches on each platform, the “other animal” category was used to ensure the avoidance of publications on human biology and medicine. For this review, we focused on articles published between 1 January 2014 and 1 April 2025. During the search process we also included early access articles in the year 2025, this was undertaken to ensure that relevant and state-of-the-art data are presented. Searching for and assessment of the studies were conducted by one author.

**Figure 1:**
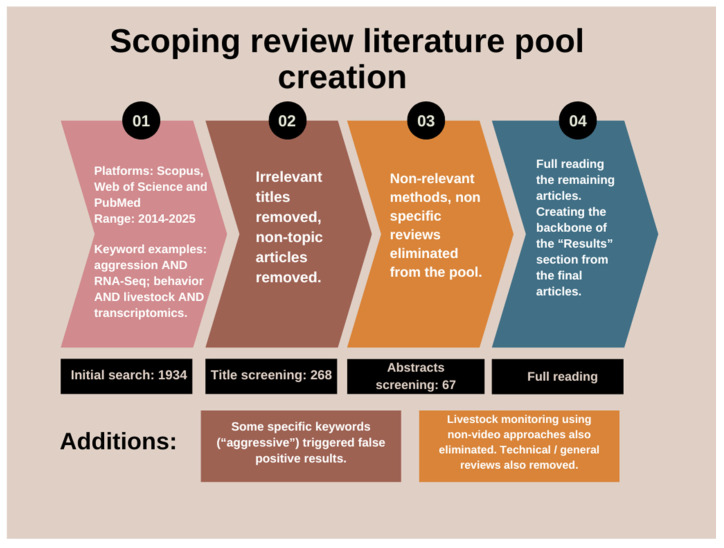
Flowchart of the main literature creation with key steps, presenting the search strategy and selection process.

Review papers were excluded from the literature pool to avoid information overlap and repetition. Studies not focusing on behavior in video analysis, or using other sensors and means of acquiring data to research other livestock-related topics were also excluded from the final library. Purely technical/method papers with no animal context were also rooted out during the screening process (Figure 1). Despite the advanced search method, there were several articles listed in the results focusing on human medicine, specifically on oncology, which were also removed. These accidental instances are attributed mainly to the keyword “aggression”.

**Figure 2:**
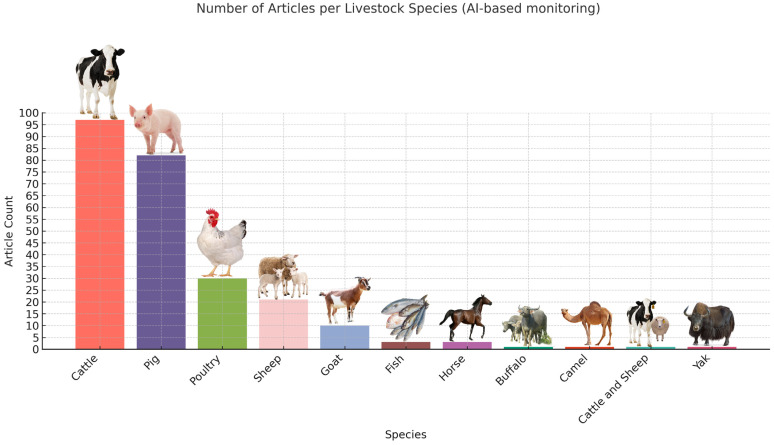
The obtained DeepLearning-based behavior monitoring articles before the final screening.

**Figure 3:**
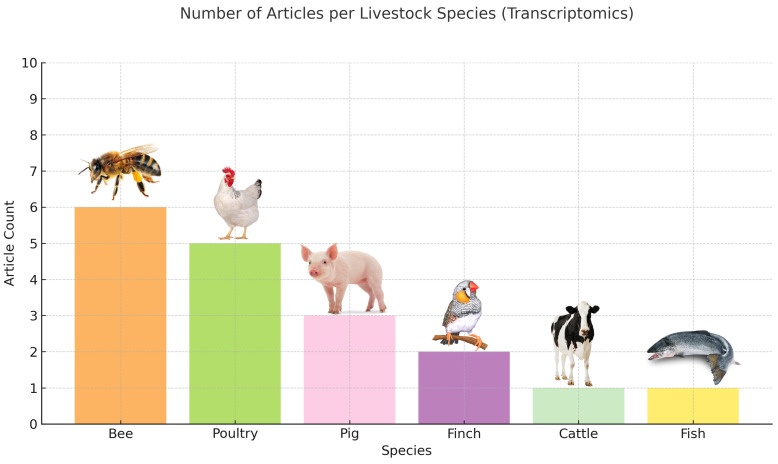
Transcriptome analysis-based behavior assessment-related articles before the final screening.

In total, 1934 articles were obtained after the initial search, combined on all three platforms. The screening method was a manual Excel (Microsoft Corporation, 2016) sorting based on the articles’ titles. After the first selection process 268 articles were obtained (250 in computer vision in livestock behavior (Table S1), 18 in transcriptomic studies in livestock behavior (Table S2) and 0 combined), thus creating the final literature pool for the scoping review (Figure 2 and Figure 3). After creating the library, a further screening was conducted by reading the abstract, material and methods, and conclusion segments of the articles. Via this approach, articles were selected for full-read analysis and creating the main content and backbone of the results segment of the review. After this further narrowing, we considered 50 articles pertaining to AI-based behavior analysis and 17 referring to transcriptomics to be key articles. During selection, behavior, social dynamics and aggression were in the focus on both subjects.

## 3. Results

### 3.1. Deep Learning-Based Video Analysis of Cattle Behavior

Deep learning techniques help precision livestock farming through automatic behavioral identification and categorization of cattle by analyzing videos. This section presents current advancements in computer vision models which detect cattle behaviors.

Multiple research projects employed object detection architectures to measure and locate cattle behaviors with exact precision. The You Only Look Once version 8 (YOLOv8) framework has received an enhanced version which integrates feature attention mechanisms with squeeze-and-excitation blocks to detect eating and lying and standing and walking behaviors effectively. The model achieved a mean average precision (mAP) of 88%, showing versatility across changing environmental conditions [10]. Another YOLOv5-based method identified both estrus-linked mounting events and also offered individual recognition of the involved animals. The two-stage process started with mounting detection, followed by region of interest-based identity recognition and produced an excellent detection accuracy of 99% [11]. The YOLOv7-E6E model, which was presented in [12] used software, including AutoAugment and GridMask, as enhancements for object detection. The data augmentation method improved the mean average precision (mAP) from 88.2% to 93.0%, thus proving the effectiveness of this approach. The researchers modified faster regions with convolutional neural network (R-CNN) features to analyze a custom cattle dataset for detecting standing and lying and walking behaviors in barn environments, achieving respective behavior-specific accuracies of 94%, 92% and 89% [13]. Another study focused on behavior action classification instead of object detection to analyze the temporal patterns of cattle activities. The approach added the flow-based equalization (FlowEQ) transform, which enhances video signals by adding motion data to an action classifier, leading to 91.5% classification accuracy and an 8% performance gain over baseline models [14]. Another system used a 3D CNN together with convolutional long short-term memory (ConvLSTM) to successfully detect both space and time-based information to organize feeding, grooming, walking, standing, and exploring behaviors. The proposed framework reached 90.32% accuracy for calf videos and 86.67% accuracy for cow videos, which outperformed state-of-the-art models, including BiLSTM and Inception-V3 [15]. Fuentes et al. [16] applied their hierarchical behavior recognition study to 15 categorical behaviors by using both instance-level and spatio-temporal aspects in RGB videos from multiple farm installations that included day and night recordings. The system integrated DeepLabCut pose estimation with LSTM networks to perform drinking behavior classification against non-drinking behavior classification. The researchers tracked essential body parts, including head, ear and neck, before they attributed the resulting temporal data to the LSTM classification model, which achieved 97.35% accuracy on test data [17]. A CNN–RNN hybrid model performed the identification of drinking and grazing behavioral patterns. The two models were calibrated before the resulting model achieved 84.88% accuracy with strong precision to demonstrate the importance of spatial and sequential feature modeling [18]. The dual-branch temporal excitation and aggregation with frequency channel attention (DB-TEAF) presented a new architecture by which to identify fighting, mounting, and running as abnormal behaviors in group-housed cattle. The model used both a motion-attentive sampling approach and a spatio-temporal attention mechanism. The system delivered enhanced precision and performance after acquiring new data because of its practical application in animal behavior and welfare monitoring [19]. The behavior recognition system of Bello et al. [20] utilized Mask R-CNN in real-time to assess four basic behaviors, which included eating, drinking and active and inactive states. The model achieved an accurate detection at 20 frames per second (FPS) while maintaining high accuracy in all categories, which makes it suitable for practical farm use. The research demonstrates an increasing preference toward real-time cattle monitoring systems that use deep learning for behavior analysis. On the other hand, behavior overlaps and group housing occlusions continue to be challenges, as shown by Fuentes et al. [21] and limited availability of complex behavior annotations in datasets are also problematic. Another research study presents a powerful surveillance system that monitors cattle behavior in real time in barn facilities through deep learning-based action detection and pose estimation. The system uses synchronized footage from four viewpoints to track hierarchical behaviors and positioning, which it then combines into a bird’s eye view (BEV) projection for unobstructed top-down visualization. The action detection model achieved state-of-the-art performance in behavior classification and spatial localization through its training on 6398 images and more than 67,000 annotations from a custom multi-camera cattle action dataset, which resulted in a mean average precision (mAP) of 99.2% and an F1-confidence peak of 98% [22].

### 3.2. Deep Learning-Based Video Analysis of Pig Behavior

Artificial intelligence-powered automated video analysis of pig behavior has become an essential tool for precision livestock farming which provides extensive solutions to welfare problems and aggression detection, reproductive management and environmental enrichment. The latest research on computer vision-based behavior recognition for pig monitoring includes aggressive and socially significant actions in pig behavior analysis.

The most difficult welfare and economic problems in pig production occur due to aggression in group-housed systems. Modern research presents multiple solutions for precise aggressive event detection. A CNN–RNN framework successfully identified tail-biting behavior with 96.25% classification accuracy together with 92.71% localization accuracy. A tracking-by-detection method with group communication reduction through pairwise contact analysis allowed early intervention possibilities according to [23]. The combination of CNN and gated recurrent unit (GRU) networks with a spatio-temporal attention mechanism enabled 94.8% accurate aggressive episode detection across 5530 labeled video segments [24]. Other authors developed an aggression analysis framework that utilizes RGB and optical flow methods with dual-attention fusion. The model detected aggression events, including their start and durations, with 68.0% average precision, which is a very important behavioral data [25]. The research conducted by Ji et al. [26] integrated temporal shift modules (TSMs) into ResNeXt50 CNNs (residual network) to achieve a 95.65% F1-score and real-time inference at 29 ms per frame. The research of Odo et al. [27] developed an efficient system that combined YOLOv4 and YOLOv7 with deep simple online and real-time tracking (DeepSORT) and centroid architectures to track and measure ear biting aggression events. Although detection reached 98% accuracy it faced tracking difficulties because false alarms reached 34%. In a different study, authors built a dual-stream faster R-CNN model to enhance automated sow posture recognition using combined RGB and depth (RGB-D) video data. The system achieved superior results when compared with traditional RGB models through depth camera usage, particularly when lighting conditions change. The approach does not address aggression directly, yet it enhances behavior monitoring reliability through day–night behavioral analysis in intensive farming systems [28]. Welfare assessment requires positive social interactions and playful behaviors as key elements. The combination of CNN-based detection with graph convolutional network (GCN) enabled the identification of preweaning piglet social interaction behaviors like nosing and seemingly aggressive play. The approach demonstrated precision of 96.6% and F1-score of 0.9535 [29]. A CNN–SVM system for detecting mating and aggressive interactions in videos achieved F1-scores of 0.9494 when tracking short durations and 0.9377 when tracking entire days [30]. The Dual-YOLOX-Tiny-ByteTrack method operated as a deep learning video system to track individual pigs in group housing environments. Through improved detection models for multi-target tracking the system delivered 98.3% detection accuracy and 97.1% tracking precision on 180,321 images. The system enabled non-invasive behavior tracking of pigs in swine production facilities without focusing on hostile conduct [31]. The detection of playing actions through a CNN–LSTM model achieved more than 92% accuracy which demonstrates computer vision applications for social dynamics detection in scalable and working systems that may extend to hostile social events, as shown in [32]. Research on general behavior recognition has also analyzed multiple types of activities, the YOLOX + SCTS-SlowFast system used object detection with temporal-spatial attention to classify eating, drinking, standing and walking behaviors, achieving 80.05% mAP and supporting full-day observations of group-housed pigs [33]. A system using fully convolutional networks (FCNs) for spatial segmentation and motion tracking detected lactating sow behaviors that included feeding and nursing along with inactivity. The model demonstrated 97.49% accuracy in drinking detection and 88.09% accuracy in nursing detection [34]. Posture detection accuracy in farrowing environments reached 98.3% when ResNet-18 used RGB images for training, because multimodal inputs proved better than RGB-only models for efficient body position classification [35]. A specialized model consisting of SlowFast + hidden Markov model performed precise sow nursing segmentation by identifying three sub-behavior patterns. The architecture reached 90.5% accuracy for behavior detection and achieved 87.05% accuracy in behavior transition identification [36]. A YOLOv8 + observation-centric SORT (OC-SORT) pipeline provided real-time behavior and identity tracking with multi-object tracking accuracy at 99.7% for long-duration videos, as the tracking ID system establishes behavioral episode connections which, according to Tu et al., provide the foundation for early welfare monitoring [37]. An enrichment engagement (EE) analysis using InceptionV3 + LSTM achieved 97.9% accuracy in differentiating between EE and non-EE clips for 1 s intervals. The authors also established object preferences [38]. In another study the dual-stream deep mutual learning network based on RGB + flow achieved 96.52% general behavior classification accuracy while maintaining low parameter complexity and short training time, which shows promise for developing scalable farm-level systems [39]. A YOLOv7 and YOLOv9 model combined with the mixed-efficient layer aggregation network (ELAN) model that used diverse convolution kernel sizes (MixConv) to detect crushing and lying behaviors, reached 0.805 mean average precision for newborn safety monitoring [40]. The implementation of deep learning-based video analysis for pig behavior monitoring has resulted in improved precision for aggression and social interaction detection as well as posture and enrichment use assessment. These systems provide scalable real-time solutions for welfare and management improvements. Although seemingly, out of all other livestock species, aggression investigation is the most advanced in pig farming, the models primarily identify single behaviors within limited contexts without considering age groups in their analysis.

### 3.3. AI-Augmented Video Analysis in Poultry

The application of AI-based video surveillance in poultry farming has developed significantly, with increasing focus on detecting behaviors through non-invasive methods. Seven studies focused on poultry, mainly on chickens and quails demonstrating diverse advancements and behavioral focuses, meaning a promising foundation for behavior monitoring in commercial poultry production. Several studies employed deep learning object detection architectures, such as different versions of YOLO models, faster R-CNN, EfficientDet, and SSD, often combined with tracking algorithms like the Kalman filter, DeepSORT, or BoT-SORT, to detect and follow poultry in their environments. Notably, ChickTrack used a multiscale-adapted YOLOv5 pipeline for detecting chickens under occlusion and changing lighting conditions, achieving reliable real-time tracking of behaviors like perching, walking and foraging, which are key signs of positive welfare [41]. Other models focused on quail behavior in caged environments. As has been shown, the I-QAMS system has integrated YOLOv7 with DeepSORT to monitor feeding and movement, achieving over 90% accuracy [42]. Similarly, a comparative study tested four architectures for detecting eating, drinking, and walking, with YOLOv5 reaching the highest precision of 85.52%, showing its effectiveness for behavior classification tasks [43]. Beyond object detection, some studies incorporated temporal modeling to assess behavior. A YOLOv8-based head detection model combined with LSTM prediction enabled group-level estimation of stress-linked deviations in cage environments by quantifying head emersion as a signal for alertness [44]. Silvera et al. [45] investigated vision-based movement analysis to assess lameness in broilers, correlating camera-derived post-disturbance activity with traditional posture scores. This study validated automated video analysis for detecting movement problems across flocks from multiple commercial farms in Europe. In a cage-free context, the use of BoT-SORT tracking embedded in YOLOv8 achieved the classification of running, exploring, and resting behaviors in layers, providing movement-based time means. The model achieved a mean average precision of 98.5%, demonstrating its effectiveness in behavior segmentation and supporting environmental comparisons between aviary setups [46]. While aggression-specific behaviors were not directly studied in most cases, several systems, like ChickTrack and BoT-SORT, lay the technical groundwork for such integration by enabling identity-aware multi-bird tracking, interaction mapping, and spatial-temporal behavior modeling. The relatively lower precision in single shot multibox detector (SSD)-based systems (60.4%) highlight the need for identity tracking developments, especially when analyzing intersecting individuals [47]. Another study introduced the fast chicken aggressive behavior recognition model (FCTR) as a transformer-based architecture for the simultaneous aggressive behavior detection and individual identification of free-range chickens through video monitoring. The system combines wearable ID tagging technology with the FCTR model to achieve precise behavioral measurement through its ability to link behavior with individual identity. The model achieved excellent performance results on the ChickenFight-2024 dataset by reaching mAP scores of 89.81% for fight detection, 85.76% for tread detection, 90.14% for peck detection and high recognition accuracy for eating and drinking behaviors. The system achieved 94.88% accuracy in individual identity matching while maintaining an overall mAP of 77.39% for identity classification. The system demonstrates its ability to detect aggression at high resolution while maintaining real-time individual tracking in farm environments [48]. Collectively, these studies confirm the viability of deep learning-based video analysis for behavior recognition and welfare monitoring in poultry, with notable success in movement, feeding, and stress-related scopings. The diversity in species (broilers, layers, quails), housing systems (cages, cage-free, aviaries), and behavioral focus (movement, rest, alertness) reflects a maturing field with strong introductory potential into aggression detection and welfare evaluation in other avian livestock species, like squab pigeon, turkey, or waterfowl species.

### 3.4. AI-Augmented Video Behavior Analysis in Minor Livestock and Aquatic Species

Research has primarily investigated AI-driven livestock behavior analysis on cattle, poultry and pigs, though scientists have also recently studied small ruminants (sheep, goat), camels, yaks, horses and fish. The scientific literature demonstrates how computer vision adapts to different environmental and production conditions by showing its relevance in challenging situations where traditional observation methods fail. This section includes four investigations about sheep which utilize deep learning to classify multiple behaviors and determine temperament and optimize technical operations. A study on multi-camera synchronization developed a timestamp-based adjusting method to ensure accurate behavior tracking across views, an essential infrastructure step for future multi-angle AI analysis [49]. The use of optical flow in combination with CNN and LSTM models by Bati & Ser [50] led to 100% accuracy in detecting fear-related temperament traits during isolation tests through motion-based inputs, versus 70% accuracy with raw frames. The results confirm stress evaluation methods while supporting genetic selection programs. A two-stage sheep behavior detector reached >98% mean average precision in detecting two natural behaviors (feeding and lying) and three disruptive actions (attacking, biting and climbing) by using a lightweight visual geometry group (VGG)-based system suitable for farm implementation. Gu et al. [51] have presented one of the rare investigations which directly focuses on aggression behavior. Other authors deployed video–acoustic equipment secured to animal halters to observe grazing patterns, browsing behaviors and chewing actions within eight hours of free-range sheep activity. This research establishes a fundamental basis for wearable monitoring devices which use multiple sensory inputs despite lacking artificial intelligence modeling [52]. The existing methods for tracking identities at scale and extended aggression observation in open pastures require improvement, though a strong foundation exists for conflict analysis. In goat behavior analysis, the GSCW-YOLO model, which employs YOLOv8n, GCT, content-aware reassembly of features (CARAFE) and Wise-IoU achieved 97.5% mean average precision and 94.1% recall while processing 9213 annotated frames at 175 FPS. The model excels at detecting minor atypical behaviors within intricate situations [53]. The second study focusing on goats implemented YOLOv4 with spatial-temporal tracking through a top-side video angle to identify eating and drinking behaviors and active and inactive activities in real-time, with a precision of ~97% [54]. Accurate real-time classification of goat behavior emerges from these studies while future research should focus on developing models for social antagonism and identification. Researchers have developed an enhanced SlowFast model with YOLOX to detect sleeping activities alongside eating and three recumbency positions in horses kept in stalls. The model demonstrated a high accuracy level of 98.77% for eating observations, followed by 93.56% for sleeping observations and a combined accuracy of 93.90% for continuous welfare monitoring of horses [55]. An AI-based camel study presented YOLOv11pose-Camel as a Bactrian camel-specific system which incorporated efficient channel attention (ECA), SimSPPF, and DECA blocks for better global feature extraction. The system achieved 94.5% accuracy together with 94.1% mean average precision to provide a powerful posture-based welfare tracking method for visually comparable species [56]. A pioneering yak behavior model achieved 96.6% total accuracy through the use of 3D ResNet50 and SlowFast to detect eating and lying and standing and walking behaviors in outdoor conditions. The system enables frame-level classification, which proves essential for ongoing health monitoring of extensive grazing systems [57]. Two research papers have contributed to computer vision applications in aquaculture. The dual-stream recurrent network (DSRN), consisting of spatial and 3D motion networks combined with LSTM, achieved 80% accuracy for feeding versus non-feeding detection in salmon within commercial environments [58]. The Aqua3DNet system achieved 80.6% accuracy and 87.3% F1-score using monocular video and salient object detection (SOD)-based depth estimation to produce 3D density heat maps of fish within dynamic environments, which benefits both welfare surveillance and feeding optimization [59]. These research projects analyze specific visual traits of aquatic animals to establish the foundation for vision-based neuroethology applications throughout aquaculture operations.

**Figure 4:**
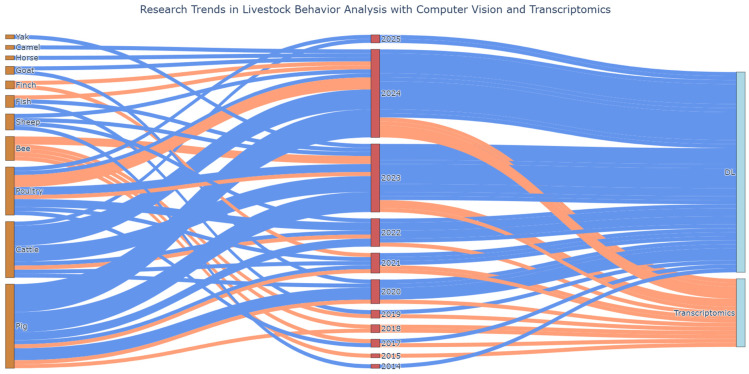
Sankey diagram presenting research trends according to focused species and year in relation to computer vision (blue strings) and transcriptomics (orange strings).

The growing range of AI-enhanced video analysis applications in various livestock sectors is demonstrated through these research studies (Figure 4), showing great versatility in species and the used models for object detection, keypoint estimation/pose estimation, behavior classification, and segmentation, sometimes even using custom frameworks (Table 1). The classification accuracy of several systems is high but there are still unexplored areas regarding aggression detection and more research is needed to model animal interactions and to track multiple animals.

### 3.5. Transcriptomic Insights into Aggression and Social Behavior in Livestock and Model Species

Knowledge of molecular mechanisms in relation with aggression in livestock helps to improve animal welfare outcomes and production efficiency and to resolve ethical issues in animal husbandry. Transcriptomic technologies now provide unique insights into the regulatory networks that manage social behaviors and even aggression. This segment presents findings from nineteen transcriptomic research projects conducted on cattle, pigs, poultry, honeybees, fish, and avian subjects which reveal vital pathways and species-specific data in neuroethology. The review also discusses and identifies research limitations, and possible solutions in developing the field.

Starting with cattle, only one research find seemed fitting in the scope of the review. Analyzing the prefrontal cortex of Lidia bulls showed major transcriptomic variations related to age, which pointed to glutamate receptor and synaptogenesis pathways as causes of aggression. Multispecies analysis confirmed overlap with known aggression-related genes, supporting the aggression mechanisms across mammals [60].

Numerous research projects have analyzed the transcriptomic data of pigs to understand their behavioral patterns. Research on weaning effects on amygdala transcriptomes demonstrated sex-specific and treatment-based gene expression changes which affected neurodevelopmental aggression-related genes [61]. Scientists studied domestic pig aggression reduction through Illumina RNA-seq analysis of wild boar and Rongchang pig frontal cortex transcriptomes. The researchers found more than 1200 genes that expressed exclusively in the study while noting major differences in behavioral regulation pathways connected to immune functions. The analysis revealed seven domestication-related genes which matched previous studies indicating a persistent tameness signal in their transcriptomes. Brain gene expression underwent significant changes due to domestication according to a study which focused on social behavior-controlling regions [62]. Researchers demonstrated how omics methods enable the discovery of stress response and damaging behavior biomarkers through their examination of complex stress-related behaviors [63].

Poultry aggression displays itself through different behavioral patterns, with aggressive pecking representing one of the main forms. Researchers combined transcriptomic and metabolomic analysis of Sansui ducks to identify eight potential genes responsible for neurodegenerative imbalance and excitatory changes which trigger aggressive pecking behavior [64]. Stress response from novelty exposure produced distinctive transcriptomic patterns in individual laying ducks. The study showed that *EXOSC3* functions as a molecular regulator of productivity traits by participating in neural communication pathways [65]. Selective breeding of Henan gamecocks for combativeness revealed excessive serotonergic–dopaminergic system activation alongside suppressed neuroimmune functions, which demonstrates an immune–aggression tradeoff. The core pathways identified through multi-omics integration consisted of tyrosine metabolism together with neuroactive components [66]. Multiple breeds underwent genomic screening, which identified aggression-related loci including *SOX5* and *DGKB* alongside *KCNMA1* that linked to muscularity [67].

Honeybees function as an excellent laboratory system to decode genomic reactions to social stimuli. One study has demonstrated that brain mushroom bodies show different genetic responses to agonistic and cooperative social interactions. Metabolic and chromosomal genes increase during aggression, which indicates that social behavior is encoded at a molecular level [68]. Under aggressive conditions, aerobic glycolysis occurs which was previously linked to cancer metabolism, but which researchers now show enhances neural activity in bees [69]. Academics observed transcriptomic changes which occurred without large chromatin alterations, indicating that specific balanced gene areas function for rapid activation [70]. Scientists analyzed aggressive behavior in worker honeybees by performing RNA sequencing experiments on their brain tissue, body fat and midgut samples. Analysts evaluated aggression connections to immunity and stress resistance by comparing gene expression between bees from high-aggression and low-aggression colonies. Evidence indicates that immune pathways show increased gene expression in non-neural tissues when linked to aggression-related genes. The findings indicate that aggression exists as a typical transcriptomic condition that emerges from social interactions [71]. The genetic variations present in colonies affect how genes related to aggression express themselves, especially in olfactory sensors [72]. The last relevant study indicates that intragenomic conflict and the allele expression stemming from parental genes lead to aggressive behaviors in animals [73].

A study on Chinook salmon behavior due to triploidy and probiotic diet used both gut microbiome analysis and brain transcriptomic methods. The triploid fish showed increased activity levels and higher expression of neuropeptide Y and neural development genes (*EGR1*, *NEUROD*, *SNAP25*) but probiotics did not bring back normal phenotypes because of the intricate relationship between genetic makeup and dietary factors and behavioral traits [74].

An interesting model species, the Zebra finch served as a valuable standard to study how hormones and environmental factors affect aggression. The study by Bentz et al. [75] found that elevated prenatal testosterone exposure led to increased adult aggression and resulted in gene expression and methylation changes of *BDNF*, *ADCY2*, and *PCSK2* in the hypothalamus and amygdala. The study by Riyahi et al. [76] found that increased sexual competition caused gene expression changes in the posterior pallium and optic tectum but not in the testes, showing that different brain regions have varying sensitivity to social hierarchy pressure.

These studies indicate that transcriptomics holds promise for aggression studies yet multiple essential gaps exist within the field. The research on transcriptomic behavior does not include enough data from livestock species, including goats and sheep, but shows a trend of increasing publications in the field on a year-by-year basis (Figure 4). The research design of the current study does not include longitudinal approaches to study transcriptomic plasticity across developmental stages and environmental cycles.

## 4. Discussion

This review addresses a major advancement in livestock behavior tracking using AI video systems, along with the transcriptomic background of aggressive behavior in domesticated animal species, but exposes fundamental gaps in research concentration, species diversity and the integration of methodologies. AI-based computer vision technologies served as the basis for 250 out of 268 studies, while transcriptomic aggression research involved only 18 investigations, without any combination of both approaches. Real-time behavioral monitoring has seen a very rapid growth in deep learning technologies for phenotyping but omics-level insights continue to progress at a slower rate. The scientific community dedicates the majority of their research to pigs and cattle for both artificial intelligence and transcriptomic studies. The majority of included studies focused on these species because their economic value and intensive production systems require precise behavior tracking tools. The research into pig group housing behavior resulted in specialized models that track tail biting and ear biting and social interactions. AI systems were widely used in cattle herds to detect mounting and standing and feeding activities. The research focuses on a few poultry studies together with sheep, goat, horse, camel and aquaculture species, which each have less than five studies. The unequal distribution of research findings creates barriers to the universal application of the research results and slows down the development of welfare monitoring systems for minor livestock species.

The reviewed studies demonstrates that AI video analysis has expanded into various livestock applications, which proves the growing adoption of this technology. Most studied systems demonstrated a detection accuracy above 90%, but their behavioral analysis remains restricted to fundamental actions such as eating, walking or lying down. The development of systems which detect social interactions as well as emotional states, reproductive behaviors and aggression under dynamic conditions continues to be inadequate. Research conducted on group-housed animals demonstrates major challenges in identifying individual animals while tracking their social interactions throughout time periods. The study of aquatic systems shows promise, but researchers have encountered specific obstacles when attempting to determine poses, depth perception and group aggression patterns in underwater conditions. Extensive livestock systems, including those associated with sheep, camels and yaks, experienced monitoring difficulties because of weather conditions, long distances and large areas that make sensor-based data collection difficult. Researchers need to develop new temporal modeling techniques, together with multi-view fusion methods and lightweight adaptive models which will support real-time applications in commercial environments and under environmental limitations.

The AI-based studies employed different technical frameworks, including CNNs, LSTMs, pose estimation and tracking algorithms. The models achieved high detection rates but worked only within specific behavioral settings, needed improved external validation and utilized datasets with different annotation practices. These models face generalization problems when moving between species groups, age classes or different farm settings. The detection of aggressive actions, together with changes in group hierarchy remains understudied through social dynamic and cross-behavior interactions.

The transcriptomic studies demonstrated valuable findings about aggression through the identification of genes involved in neurodevelopment, immune response, neuroendocrine signaling and social behavior regulation. The research focused mainly on pigs, together with chickens and honeybees, but most other livestock, such as goats, sheep and aquatic species, received minimal attention. Research on gene expression changes throughout development and environmental cycles remains non-existent in the current literature. Most of the existing research measured gene activity through static moments taken after specific events, making it difficult to align behavioral data temporally. The field lacks sufficient qPCR, CRISPR, RNAi and GWAS methods for validating candidate genes identified through transcriptomic studies, which reduces their practical applications. The research shows that transcriptomics shows excellent potential for studying aggression, but numerous and essential knowledge gaps exist. The research community should pursue the following goals: Multiple tissues need to receive integrated analysis of transcriptomics with proteomics and epigenomics, standard behavioral assays need development to establish direct relationships between molecular profiles and aggression phenotypes, and studies must establish translation methods that link findings from bee and finch models to farm animal applications.

The scoping review demonstrates that no investigations exist to combine AI behavioral monitoring with transcriptomic profiling. Computational neuroethology misses a crucial chance because the synchronization between high-frequency behavioral data and biological sampling would advance aggression research and predictive modeling. The implementation of such integration demands the development of unified data platforms and time-stamped behavioral records that match tissue samples alongside interdisciplinary research teams. We outline the following essential steps for advancing this field:

- The field requires expansion to underrepresented species in order to develop fair behavioral information across worldwide livestock systems;
- The creation of multimodal datasets should include combining AI-generated behavior tracking systems with molecular sampling systems.
- AI models require evaluation through cross-validation and benchmarking tests to determine their performance across different species and environmental settings and behavioral domains.
- There should be a standardization of behavioral definitions, annotation practices, and model performance reporting in order to support meta-analysis and reproducibility.
- The evaluation of both behavioral and transcriptomic systems in real farms should occur in order to guarantee their practical use in animal husbandry.

Future research should achieve ethical and efficient livestock systems with intelligent management of aggression through technological and biological frameworks after addressing structural and scientific challenges.

## 5. Conclusions

The combination of AI-based behavioral phenotyping with transcriptomic profiling presents substantial potential for future precision livestock farming development. The review establishes current field status while providing a strategic direction to speed up behavioral and molecular data integration. Future studies could achieve better animal behavior understanding through data-rich standardized multi-species approaches, which will lead to more ethical, productive and resilient livestock systems.

## Figures and Tables

**Table 1:** Summarized table of computer vision frameworks by species.

Species	Detection/Tracking	Classification Models	Pose/Keypoint Estimation
Cattle	Faster R-CNN Mask R-CNN YOLOv5 YOLOv7 YOLOv8	CNN–RNN ConvLSTM DB-TEAF FlowEQ LSTM	BEV DeepLabCut
Pigs	BoT-SORT DeepSORT Faster R-CNN YOLOX YOLOv4 YOLOv7 YOLOv8	CNN-GRU CNN-LSTM CNN–RNN FCN HMM ResNet-18 SVM SlowFast Transformer	3D CNN RGB-D Spatial-temporal tracking
Poultry	BoT-SORT DeepSORT EfficientDet Faster R-CNN Kalman Filter SSD YOLOv5 YOLOv7 YOLOv8	LSTM Transformer	Head emersion model Posture tracking
Minor livestock	VGG YOLOX YOLOv11 YOLOv4 YOLOv8	CARAFE CNN GCT LSTM ResNet-50 SlowFast	3D ResNet DeepLabCut SOD-based depth estimation

## Data Availability

No new data were created or analyzed in this study.

## Supplementary Materials

The following supporting information can be downloaded at https://www.mdpi.com/article/10.3390/biology14070771/s1, Table S1. List of articles on DeepLearning based livestock behavior analysis before final screening. Table S2. List of articles on transcriptomics based livestock behavior analysis before final screening.
